# Lifetime benefits of comprehensive medical therapy in heart failure with mildly reduced or preserved ejection fraction

**DOI:** 10.1038/s41591-025-04037-3

**Published:** 2025-10-06

**Authors:** Muthiah Vaduganathan, Brian L. Claggett, Safia Chatur, Akshay S. Desai, Pardeep S. Jhund, Orly Vardeny, Bela Merkely, Felipe Martinez, Josep Comin-Colet, Jose F. Kerr Saraiva, Sanjiv J. Shah, Carolyn S. P. Lam, Faiez Zannad, Kieran F. Docherty, John J. V. McMurray, Scott D. Solomon

**Affiliations:** 1 Brigham and Women’s Hospital, Harvard Medical School, Boston, MA USA; 2 BHF Glasgow Cardiovascular Research Centre, School of Cardiovascular and Metabolic Medicine, University of Glasgow, Glasgow, UK; 3 Center for Chronic Disease Outcomes Research, Minneapolis VA Health Care System, University of Minnesota, Minneapolis, MN USA; 4 Semmelweis University, Heart and Vascular Centre, Budapest, Hungary; 5 Universidad Nacional de Córdoba, Córdoba, Argentina; 6 Bellvitge University Hospital (IDIBELL, CIBERCV), Universitat de Barcelona, Barcelona, Spain; 7 Pontifical Catholic University of Campinas, Campinas, Brazil; 8 Northwestern University Feinberg School of Medicine, Chicago, IL USA; 9 National Heart Centre Singapore, Duke-National University of Singapore, Singapore, Singapore; 10 University of Lorraine, Nancy, France

**Keywords:** Heart failure, Randomized controlled trials

## Abstract

Sodium–glucose cotransporter-2 inhibitors (SGLT2i) and the nonsteroidal mineralocorticoid receptor antagonist (nsMRA) finerenone have each been shown to individually improve heart failure events among patients with heart failure and mildly reduced or preserved ejection fraction (HFmrEF/HFpEF). Moreover, the angiotensin receptor neprilysin inhibitor (ARNI) sacubitril/valsartan has been shown to improve outcomes in patients with HFmrEF/HFpEF with a left ventricular ejection fraction (LVEF) below normal (<60%). However, the expected benefits of the combined use of these agents with long-term administration are not well defined. Here, in this cross-trial analysis of DELIVER, FINEARTS-HF and PARAGON-HF, combined use of SGLT2i and nsMRA therapies was estimated to reduce the risk of cardiovascular death or first worsening heart failure event by 31% in the overall population (hazard ratio 0.69; 95% confidence interval 0.59–0.81), while combined use of SGLT2i, nsMRA and ARNI therapies was estimated to reduce risk by 39% in patients with HFmrEF/HFpEF and an LVEF <60% (hazard ratio 0.61; 95% confidence interval 0.48–0.77). With long-term use, combined SGLT2i and nsMRA therapies in a 65-year-old patient with HFmrEF/HFpEF, or combined SGLT2i, nsMRA and ARNI therapies in a 65-year-old patient with an LVEF <60%, were projected to afford 3.6 (2.0–5.2) or 4.9 (2.5–7.3) additional years free from cardiovascular death or a heart failure event, respectively. Combined therapy was estimated to result in meaningful gains in event-free survival across a broad age range, from 55 to 85 years. Among patients with HFmrEF and HFpEF, the potential aggregated long-term treatment effects of early combination medical therapy with SGLT2i and nsMRA (and ARNI in selected individuals) are projected to be substantial.

## Abstract (web summary)

In a cross-trial analysis, combined treatment with medications that have individually shown to improve cardiovascular outcomes in heart failure with mildly reduced or preserved ejection fraction demonstrates substantial benefit, including for event-free survival.

## Main

Patients with heart failure (HF) with preserved ejection fraction (HFpEF) experience life expectancies that are considerably shorter than their peers of similar ages^1^. Until recently, the management of HFpEF was largely empirical and limited to diuretics, blood pressure control and comorbidity management. Indeed, before 2023, major clinical practical guidelines offered no class I (strong) recommendation for any specific pharmacotherapy (beyond diuretics) in the treatment of HFpEF. However, since then, the sodium–glucose cotransporter-2 inhibitors (SGLT2i) have been shown to improve cardiovascular outcomes and are strongly recommended in patients with HF and mildly reduced or preserved ejection fraction (HFmrEF/HFpEF), that is, a left ventricular ejection fraction (LVEF) >40% (refs. ^2,3^). A trial of the nonsteroidal mineralocorticoid receptor antagonist (nsMRA) finerenone demonstrated its efficacy and safety in this same population, which supported its recent regulatory approval by the US Food and Drug Administration^4^. In addition, a trial of the angiotensin receptor neprilysin inhibitor (ARNI) sacubitril/valsartan, while narrowly missing its primary endpoint^5^, suggested efficacy in patients with a LVEF below normal (<60%)^6^. Smaller trials with shorter-duration follow-up periods have suggested potential early efficacy with incretin-based therapies, but long-term outcomes trials are awaited. As randomized clinical trials tested these therapies individually in trials conducted over an average follow-up of 2–3 years on the background of varying medical therapy regimens, the expected benefits of their combined use when administered long-term are not well defined.

Multiple stakeholders (patients, clinicians, health systems and payors) may be interested in the therapeutic potential of these therapies when used together in the management of this growing population. As such, we first estimated the aggregate relative benefits of SGLT2i and nsMRA in all patients with HFmrEF/HFpEF and the combination of SGLT2i, nsMRA and ARNI in those with HFmrEF/HFpEF and a LVEF <60%. We then projected the potential absolute long-term gains in event-free survival with comprehensive medical therapy.

## Results

### Relative treatment effects of comprehensive medical therapy

For the main analysis, we derived treatment estimates from 6,263 participants in DELIVER (Dapagliflozin Evaluation to Improve the LIVEs of Patients with Preserved Ejection Fraction Heart Failure) and 6,001 participants in FINEARTS-HF (FINerenone trial to investigate Efficacy and sAfety superioR to placebo in paTientS with Heart Failure) (Table 1). In the subgroup of individuals with LVEF below normal (<60%), we estimated treatment effects from 4,372 participants in DELIVER, 4,846 in FINEARTS-HF and 2,070 in PARAGON-HF (Prospective Comparison of ARNI with ARB Global Outcomes in HF with Preserved Ejection Fraction). These trials evaluated older participants with HF (mean ages 72–73 years), with balanced sex distribution (44–52% women) and a high rate of comorbidities. Background mineralocorticoid receptor antagonist (MRA) use was 26% in PARAGON-HF and 43% in DELIVER. Background ARNI use was 5% in DELIVER and 9% in FINEARTS-HF. Background SGLT2i use was 1% in PARAGON-HF and 14% in FINEARTS-HF (Table 2). Within each of the trials, serious adverse events were reported at similar frequencies between the study arms. Hypotension (systolic blood pressure <100 mmHg) was more common with finerenone (versus placebo) and ARNI (versus valsartan), and elevated serum potassium >5.5 mmol l^−1^ was more common with finerenone (versus placebo) (Table 3).

**Table 1:** Key trial design features

	DELIVER (*n* = 6,263)	FINEARTS-HF (*n* = 6,001)	PARAGON-HF (*n* = 4,796)
**Comparison**	Dapagliflozin versus placebo	Finerenone versus placebo	Sacubitril/valsartan versus valsartan
**Study type**	Randomized, double-blind, clinical trial	Randomized, double-blind, clinical trial	Randomized, double-blind, clinical trial
**Enrollment period**	2018–2021	2020–2023	2014–2016
**Global enrollment**	350 sites in 20 countries	654 sites in 37 countries	848 sites in 43 countries
**Median follow-up (years)**	2.3	2.7	2.9
**Patient population**	Patients ≥40 years with HF with NYHA class II–IV functional class symptoms	Patients ≥40 years with HF with NYHA class II–IV functional class symptoms	Patients ≥18 years with HF with NYHA class II–IV functional class symptoms
**Setting of enrollment**	Patients could be randomized across ambulatory and hospitalized populations	Patients could be randomized across ambulatory and hospitalized populations	Patients could be screened, but not randomized during hospitalization for HF
**LVEF**	>40%	≥40%	≥45%
**Cardiac structure and function**	Evidence of structural heart disease (that is, left ventricular hypertrophy or left atrial enlargement)	Evidence of structural heart disease (that is, left ventricular hypertrophy or left atrial enlargement)	Evidence of structural heart disease (that is, left ventricular hypertrophy or left atrial enlargement)
**Natriuretic peptides**	NT-proBNP ≥600 pg ml^−1^ (in AF) or NT-proBNP ≥300 pg ml^−1^ (if not in AF)	NT-proBNP ≥300 pg ml^−1^ (or BNP ≥100 pg ml^−1^) within 30 days (in those without a recent worsening HF event) or within 90 days (in those with a recent worsening HF event). Qualifying levels of NT-proBNP or BNP were tripled in AF	NT-proBNP >900 pg ml^−1^ (in AF) or NT-proBNP >300 pg ml^−1^ (if not in AF) If recently hospitalized for HF within 9 months, NT-proBNP >600 pg ml^−1^ (in AF) or NT-proBNP >200 pg ml^−1^ (if not in AF)
**Body mass index**	≤50 kg m^−^^2^	≤50 kg m^−^^2^	≤40 kg m^−^^2^
**Systolic blood pressure**	≥95 mm Hg at screening and at randomization	≥90 mm Hg at screening and at randomization	≥110 mm Hg at screening and ≥100 mm Hg at randomization
**Potassium**	–	≤5.0 mmol l^−1^ at screening and at randomization	≤5.2 mEq l^−1^ at screening and ≤5.4 mEq l^−1^ at randomization
**eGFR**	≥25 ml min^−1^ per 1.73 m^2^ at screening	≥25 ml min^−1^ per 1.73 m^2^ at screening and at randomization	≥30 ml min^−1^ per 1.73 m^2^ at screening and ≥25 ml min^−1^ per 1.73 m^2^ at randomization and without greater than a 35% reduction in eGFR during either run-in period
**Run-in period**	None	None	Single-blind run-in phase with half-target doses of both study drugs
**Primary Endpoint**	Cardiovascular death or first worsening HF event	Cardiovascular and total worsening HF events	Cardiovascular death and total HF hospitalizations

AF, atrial fibrillation/flutter; BNP, B-type natriuretic peptide; eGFR, estimated glomerular filtration rate; NT-proBNP, N-terminal prohormone of B-type natriuretic peptide; NYHA, New York Heart Association.

**Table 2:** Selected baseline characteristics

	DELIVER (*n* = 6,263)	FINEARTS-HF (*n* = 6,001)	PARAGON-HF (*n* = 4,796)
**Age (years)**	72 ± 10	72 ± 9	73 ± 8
**Women**	2,747 (44%)	2,732 (46%)	2,479 (52%)
**Body mass index, kg** **m**^**−**^^**2**^ **mean** **±** **s.d.**	30 ± 6	30 ± 6	30 ± 5
**Systolic blood pressure (mm** **Hg), mean** **±** **s.d.**	128 ± 15	129 ± 15	131 ± 16
**LVEF (%), mean** **±** **s.d.**	54 ± 9	53 ± 8	58 ± 8
**New York Heart Association class III or IV**	1,549 (25%)	1,854 (31%)	951 (20%)
**Atrial fibrillation**	3,552 (57%)	3,273 (55%)	2,521 (53%)
**Diabetes mellitus**	2,806 (45%)	2439 (41%)	2,062 (43%)
**Loop diuretics**	4,811 (77%)	5239 (87%)	3,757 (78%)
**ARNI**	301 (5%)	513 (9%)	–
**SGLT2 inhibitor**	–	817 (14%)	28 (0.6%)
**MRA**	2,667 (43%)	–	1,239 (26%)
**β-Blockers**	5,167 (83%)	5,095 (85%)	3,821 (80%)

Data are presented as *n* (%) or mean (s.d.), unless otherwise stated.

s.d., standard deviation.

**Table 3:** Selected adverse events

Safety event	DELIVER		FINEARTS-HF		PARAGON-HF	
	Dapagliflozin	Placebo	Finerenone	Placebo	Sacubitril/valsartan	Valsartan
**Any serious adverse event**	1,361/3,126 (43.5)	1,423/3,127 (45.5)	1,157/2,993 (38.7)	1,213/2,993 (40.5)	1,424/2,419 (58.9)	1,416/2,402 (59.0)
**Renal impairment**	10/3,126 (0.3)	7/3,127 (0.2)	57/2,897 (2.0)	34/2,888 (1.2)	38/2,407 (1.6)	40/2,389 (1.7)
**Hypotension**	6/3,126 (0.2)	1/3,127 (0.0)	538/2,911 (18.5)	361/2,904 (12.4)	380/2,407 (15.8)	257/2,389 (10.8)
**Elevated serum potassium** >**5.5** **mmol** **l**^**−1**^	–	–	413/2,898 (14.3)	199/2,889 (6.9)	316/2,386 (13.2)	361/2,367 (15.3)

Adverse events were defined differently across trials and were collected in the safety analytic sets. Renal impairment was defined as events leading to permanent study drug discontinuation (in DELIVER) and as serum creatinine levels ≥3.0 mg dl^−1^ (in FINEARTS-HF and PARAGON-HF). Hypotension was defined as events leading to permanent study drug discontinuation (in DELIVER), any visit systolic blood pressure <100 mm Hg (in FINEARTS-HF) and investigator-reported hypotension with systolic blood pressure <100 mm Hg (in PARAGON-HF). Serum potassium levels after randomization were not collected in the DELIVER trial.

**Fig. 1:**
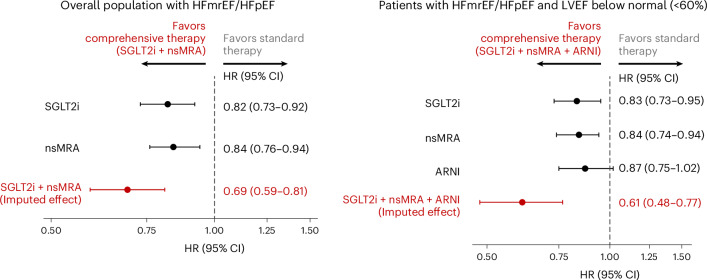
Aggregate relative risk reduction on cardiovascular death or worsening HF event with comprehensive medical therapy. Aggregate relative benefits of SGLT2i and nsMRA in the overall population (left) and the combination of SGLT2i, nsMRA, and ARNI in individuals with a LVEF below normal <60% (right). Treatment estimates for individual therapies and their combination are summarized as HRs and 95% CIs. ‘Standard treatment’ in the comparator populations constituted treatment according to the standard of care based on local guidelines, but did not mandate any specific pharmacotherapy. For the main analysis, we derived treatment estimates from 6,263 participants in DELIVER and 6,001 participants in FINEARTS-HF. In the subgroup of individuals with LVEF below normal (<60%), we estimated treatment effects from 4,372 participants in DELIVER, 4,846 in FINEARTS-HF and 2,070 in PARAGON-HF.

Combination use of SGLT2i and nsMRA was estimated to reduce the risk of the primary endpoint of cardiovascular death or first worsening HF event by 31% in the overall HFmEF/HFpEF population (hazard ratio (HR) 0.69; 95% confidence interval (CI) 0.59–0.81). In individuals with an LVEF below normal (<60%), the combined use of SGLT2i, nsMRA and ARNI was estimated to reduce risk by 39% (HR 0.61; 95% CI 0.48–0.77) (Fig. 1). When considering the effects of ARNI (against a putative placebo), the combination use of SGLT2i, nsMRA and ARNI (versus putative placebo) was estimated to reduce risk by 47% (HR 0.53; 95% CI 0.39–0.71). In the overall population, results were consistent in a sensitivity analysis examining SGLT2i as a class (based on meta-analysis of DELIVER and EMPEROR-Preserved) and MRAs as a class (based on meta-analysis of FINEARTS-HF and TOPCAT) (aggregate HR 0.70; 95% CI 0.61–0.79). In sensitivity analyses, aggregate relative treatment effects remained robust for the composite of all-cause death or worsening HF event (HR 0.76; 95% CI 0.66–0.87) to account for competing risks of mortality and when evaluating the composite of cardiovascular death or HF hospitalization (not considering urgent HF visits) (HR 0.71; 95% CI 0.60–0.84).

Applying guideline-concordant LVEF designations, comprehensive medical therapy was estimated to reduce the primary endpoint by 32% (HR 0.68; 95% CI 0.56–0.83) in those with HFpEF (LVEF ≥50%) with SGLT2i and nsMRA and by 42% (HR 0.58; 95% CI 0.40–0.85) in those with HFmrEF (LVEF 40–50%) with SGLT2i, nsMRA and ARNI.

### 3-Year absolute risk reductions and number-needed-to-treat

We estimated event-free survival in 1,754 participants with HFmrEF/HFpEF in the control arm of the DELIVER trial (main analysis) and in the subset of 1,123 participants with HFmrEF/HFpEF and an LVEF below normal (<60%). Mean age was 72.5 ± 9.0 years, and 818 (46.6%) were women (Extended Data Table 1).

In the overall HFmrEF/HFpEF population, over a median within-trial follow-up of 2.2 years (25th–75th percentiles 1.5–2.7 years), 330 primary events were observed with a corresponding event rate of 9.1 (95% CI 8.1–10.1) per 100 patient-years. Based on the observed annualized event rate in the control group of the DELIVER trial, the estimated range of aggregate absolute risk reductions over 3 years of comprehensive medical therapy was 4–9%, corresponding to a number-needed-to-treat of 11–25 to prevent one primary endpoint event.

In the LVEF-below-normal subpopulation, over a median within-trial follow-up of 2.3 years (25th–75th percentiles 1.5–2.8 years), 211 primary events were observed with a corresponding event rate of 9.1 (95% CI 7.9–10.4). With 3 years of comprehensive medical therapy, the absolute risk reductions would range from 5% to 12%, corresponding to a number-needed-to-treat of 9–20 to prevent one primary endpoint event.

### Long-term projections of event-free survival gains

**Fig. 2:**
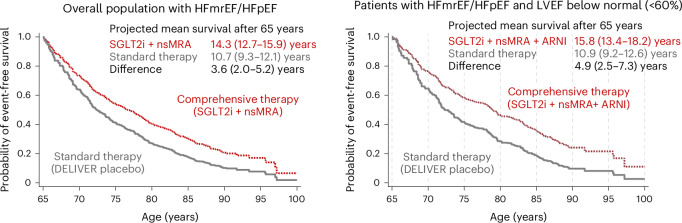
Estimated absolute event-free survival gains with comprehensive medical therapy. Kaplan–Meier estimated curves for patients starting at 65 years of age for survival free from the primary endpoint (cardiovascular death or worsening HF event) in the overall HFmrEF/HFpEF population (left) and in individuals with HFmrEF/HFpEF with a LVEF below normal <60% (right). Residual event-free lifespan was estimated using the area under the survival curve up to a maximum of 100 years of age.

**Fig. 3:**
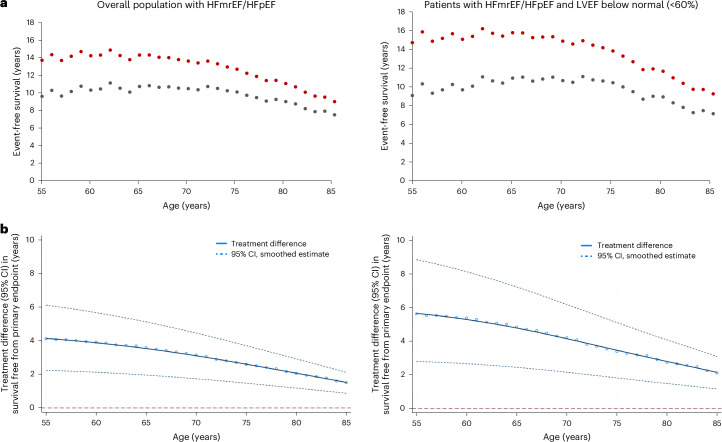
Event-free survival gains with comprehensive medical therapy across the age spectrum. **a**, Estimated mean survival free from the primary endpoint in the DELIVER control group and the simulated comprehensive medical therapy group for every age between 55 and 85 years in the overall population with HFmrEF/HFpEF (left) or patients with HFmrEF/HFpEF and LVEF below normal (<60%) (right). **b**, Treatment differences (data points), smoothed estimates (solid lines) and 95% CI of the smoothed estimates (dashed lines) are displayed for mean event-free survival with comprehensive medical therapy after application of a locally weighted scatterplot smoothing procedure. Data are shown for the overall population with HFmrEF/HFpEF (left) or patients with HFmrEF/HFpEF and LVEF below normal (<60%) (right).

In the overall population, forecasted long-term survival free from the primary endpoint (cardiovascular death or worsening HF event) was estimated to be 10.7 years (95% CI 9.3–12.1) in placebo-treated participants on standard therapy and 14.3 years (95% CI 12.7–15.9) with comprehensive treatment with SGLT2i and nsMRA. We estimated comprehensive medical therapy to provide 3.6 (2.0–5.2) additional years free from cardiovascular death or HF event in a 65-year-old participant (Fig. 2). Event-free survival gains remained substantial in sensitivity analyses assuming subadditive treatment effects of therapies (Extended Data Table 2) and waning efficacy of comprehensive medical therapy over time (Extended Data Table 3). Meaningful gains in event-free survival were observed across a broad age range (Fig. 3) from 1.5 (0.9–2.1) additional years in an 85-year-old to 4.1 (2.2–6.1) additional years in a 55-year-old. In a 65-year-old HFmrEF/HFpEF patient with LVEF below normal (<60%), comprehensive treatment with SGLT2i, nsMRA and ARNI was estimated to afford 4.9 (2.5–7.3) years free from cardiovascular death or HF event.

## Discussion

The global prevalence of HFpEF is projected to continue rising^7^, and individuals living with the disease have a guarded prognosis and face a high burden of hospitalizations and healthcare encounters^1^. As such, extending event-free survival represents a core treatment goal in this high-risk population. In the adjacent population of HF with reduced ejection fraction, we previously estimated that comprehensive medical therapy with four therapies (targeting five distinct pathways) with ARNI, β-blocker, MRA and SGLT2i could afford over 6 years of additional event-free survival in a 65-year-old compared with conventional medical therapy (consisting of an angiotensin-converting enzyme inhibitor or angiotensin receptor blocker and β-blocker)^8^. This ‘pillar-based’ comprehensive medical therapy approach has now been embraced by contemporary clinical practice guidelines and represents the new standard of care globally for HFrEF^9,10^.

HFpEF is recognized to be a systemic syndrome with both myocardial dysfunction and peripheral abnormalities that contribute to disease progression^11^. Similar to HFrEF, combining multiple therapies targeting distinct pathophysiologic mechanisms may most comprehensively attenuate risks of morbidity and mortality. The SGLT2i represented the first class of therapies (beyond diuretics) that have been strongly recommended as a class I guideline recommendation across the full spectrum of LVEF, including in HFpEF^9^. The recent FINEARTS-HF trial^4^ demonstrated that a second therapy, the nsMRA finerenone, is beneficial in improving outcomes in patients with HFmrEF/HFpEF, including among those already treated with an SGLT2i. The US Food and Drug Administration has since approved finerenone for the management of patients with HF and an LVEF ≥40%. The ARNI sacubitril/valsartan, which targets both the renin–angiotensin system and the natriuretic peptide axis, appears to be most beneficial in those with LVEF below normal^5,6^ and is now approved for use in many countries worldwide for this indication.

Compared with standard care (which encompassed management of congestion and comorbidities such as hypertension), comprehensive medical therapy inclusive of SGLT2i and nsMRA was estimated to reduce risks of cardiovascular death or worsening HF events by over 30%, and the further addition of ARNI among individuals with an LVEF below normal was estimated to reduce risks of clinical events by nearly 40%. Previous cross-trial analyses were limited to estimating the relative treatment benefits with comprehensive medical therapy without offering perspective on potential absolute treatment gains, especially over a long-term horizon^12^. Over 3 years, the estimated absolute risk reduction ranged from 4% to 9%, corresponding to a number-needed-to-treat of 11–25 to prevent a clinical event. Over a lifetime horizon, we estimated that comprehensive medical therapy would substantially extend event-free survival in the overall population and in those with LVEF below normal. As younger individuals with HF have longer disease duration and expected residual lifespan, estimated gains in event-free survival with comprehensive medical therapy were greatest in this population. However, across a broad age range, including among people aged 85 years and older, we forecasted meaningful absolute gains in event-free survival. Taken together, these cross-trial data analyses summarize important therapeutic advances in recent years and serve as a reference for relative and absolute gains that might be expected with comprehensive medical therapy.

The first central assumption in our study was that each individual therapy provides additive benefits in patients with HFmrEF/HFpEF. Several lines of evidence suggest that this is an acceptable analytic assumption. First, each of the therapies evaluated has a distinct mechanism of action with no known pharmacological interactions. Second, subgroup analyses from pivotal randomized clinical trials have shown that the benefits of one therapy do not appear attenuated based on background treatment regimens^13–15^, suggesting complementary protection against clinical events. However, we acknowledge that background use of these therapies was incomplete, potentially limiting the power to detect heterogeneity if indeed it was present. Third, recent trials have directly tested the combined use of SGLT2i and MRAs in patients with HFpEF and separately in individuals with chronic kidney disease and shown that combination therapy affords incremental and additive effects on markers of cardiovascular and kidney health^16,17^. Reassuringly, even when assuming subadditive benefits of these therapies, the long-term gains in event-free survival are projected to be substantial.

The second central assumption was that the therapeutic effects observed during each trial would be sustained during lifetime use of therapies. Clinical trials of HF therapies are often conducted with average follow-up durations of 2–3 years; however, guidelines recommend the long-term continuation of these therapies for much longer treatment horizons, often indefinitely. We developed and validated a methodology to project within-trial observations to estimate long-term disease trajectories, assuming stable treatment effects over time^18,19^. However, adherence in real-world clinical care settings is known to be lower than during the conduct of clinical trials.

The clinical benefits of both SGLT2i and nsMRA have been shown to attenuate even after short-term drug interruption (within 30 days)^20,21^. However, clinically relevant gains in long-term event-free survival are expected even when assuming waning efficacy of comprehensive medical therapy over time. However, we did not consider other critical issues regarding the long-term use of therapies, including costs, ongoing access, treatment complexity and polypharmacy. These factors should be considered in the overall assessment of the risks and benefits of comprehensive medical therapy when applying these summary efficacy data to clinical practice.

The final central assumption is that the long-term benefits projected in this cross-trial analysis would translate to clinically relevant benefits when implemented in ‘real-world’ settings. Participants were carefully selected according to specific eligibility criteria in each trial, and in PARAGON-HF, patients were required to tolerate half-target doses of each of the study drugs before randomization^5^. We considered treatment effects derived from the overall trial populations (aside from the subpopulation with LVEF below normal) as clinical trials have not consistently demonstrated heterogeneity by individual subgroups, but effectiveness of these therapies may still vary in individual patients when applied in usual clinical care settings. Unlike the previous cross-trial analysis in HFrEF^8^, we projected long-term event-free survival gains with comprehensive medical therapy in HFmrEF/HFpEF but did not consider additive effects on mortality outcomes as none of the individual trials demonstrated significant benefits on overall or cardiovascular mortality. In light of these considerations, we intentionally made conservative analytic choices in our long-term projections and subjected our findings to a range of sensitivity analyses to support their robustness.

While we focused on the aggregate benefits that may be realized with complete implementation of these therapies, safety and tolerability cannot be ignored when considering multidrug regimens in older individuals with HFpEF. Data from pivotal trials support the safety of these therapies when initiated on the background of varying medical regimens^3–5^. In routine clinical practice, however, similar follow-up protocols with close monitoring and frequent study visits may be challenging to replicate. All three classes of therapies are hemodynamically active with potentially additive blood pressure lowering when initiated together^16,17^. Simultaneous initiation of MRAs and SGLT2i is also known to induce potentially additive acute reductions in kidney function^16,17^ that appear entirely hemodynamically mediated and not associated with tubular injury or long-term adverse prognosis. MRAs such as finerenone are known to increase serum potassium levels, but early combination with either an SGLT2i^22^ or ARNI^23^ (when switched from a renin–angiotensin system inhibitor) may attenuate risks of hyperkalemia, suggesting that these combinations may in fact be safer. It is reassuring that, upon drug cessation, the early changes in hemodynamics, kidney function and potassium are fully reversible^17,21^. Although initial data from implementation trials^24^ suggest that the rapid, sequential initiation of multidrug regimens is generally safe, a more gradual, stepwise approach may be necessary for patients predicted to have poorer tolerability (such as those who are frail or clinically unstable).

The therapeutic landscape of HFpEF continues to rapidly evolve. We attempted to consider the totality of available evidence, including major positive trials powered for clinical outcomes in broad populations of HFpEF, that have supported regulatory approvals for the management of this condition. There has been considerable interest in the potential role of obesity-targeted therapies, such as the glucagon-like peptide-1 receptor agonists and related compounds, in the management of HFpEF. In fact, three recent trials with sample sizes of approximately 500–750 participants have demonstrated clinical benefits^25–27^. However, while awaiting larger trials (such as NCT07037459), we have not considered these therapies in the cross-trial analysis given the exclusive focus on a specific phenotype of HFpEF (with a body mass index ≥30 kg m^−^^2^), limited number of clinical events (<100 events in each of the trials completed thus far) and relatively short duration of follow-up (1–2 years). Similarly, we did not consider use of renin–angiotensin system inhibitors alone, despite their common use in this population for the management of comorbidities (such as hypertension, diabetes, coronary artery disease and chronic kidney disease), as primary trials did not meet their primary endpoints^28,29^ and these therapies are not approved for this indication.

Among patients with HFmrEF and HFpEF, the anticipated aggregate long-term treatment effects of early comprehensive medical therapy with SGLT2i and nsMRA (and ARNI in selected individuals) are projected to be substantial. These data underscore the urgent need to bolster global implementation efforts to improve the use of medical therapies in HFmrEF/HFpEF, a population with previously limited therapeutic options.

## Methods

In this cross-trial analysis, we identified pivotal trials that supported the regulatory evaluation of therapies approved by the US Food and Drug Administration (as of August 2025) for the management of HFmrEF/HFpEF. We leveraged individual participant-level data that we had direct access to to represent each class of therapies: SGLT2i (dapagliflozin in DELIVER), nsMRA (finerenone in FINEARTS-HF) and ARNI (sacubitril/valsartan in PARAGON-HF). Table 1 summarizes key design features of each trial. All trials were assessed as high quality with a low risk of bias (Extended Data Table 4). The primary results of each trial have been previously published, and the study protocols and statistical analysis plans are publicly available^3–5^. All participants provided informed consent, and trial protocols were approved by local institutional review boards or ethics committees.

### DELIVER

From 2018 to 2021, the DELIVER (ClinicalTrials.gov Identifier: NCT03619213) trial^3^ randomly assigned 6,263 adults ≥40 years with symptomatic HF and an LVEF >40% to dapagliflozin 10 mg once daily or matching placebo. All participants were required to have evidence of structural heart disease and elevated natriuretic peptide levels. Median follow-up was 2.3 years.

### FINEARTS-HF

From 2020 to 2023, the FINEARTS-HF (ClinicalTrials.gov Identifier: NCT04435626) trial^4^ randomly assigned 6,001 adults ≥40 years with symptomatic HF and an LVEF ≥40% to finerenone or matching placebo titrated to target doses of 20 mg or 40 mg (depending on baseline estimated glomerular filtration rate). All participants were required to have evidence of structural heart disease and elevated natriuretic peptide levels. Median follow-up was 2.7 years.

### PARAGON-HF

From 2014 to 2016, the PARAGON-HF (ClinicalTrials.gov Identifier: NCT01920711) trial^5^ randomly assigned 4,796 adults ≥18 years with symptomatic HF and an LVEF ≥45% to the ARNI sacubitril/valsartan (target dose, 97 mg of sacubitril with 103 mg of valsartan twice daily) versus the angiotensin receptor blocker valsartan (target dose, 160 mg twice daily). Only participants who tolerated half target doses of both study medications during a single-blind run-in phase were randomized. All participants were required to have evidence of structural heart disease and elevated natriuretic peptide levels. Median follow-up was 2.9 years.

### Clinical outcomes

Our primary endpoint was a composite of cardiovascular death or worsening HF event (which included both a hospitalization for HF and an urgent ambulatory encounter for HF requiring intravenous HF therapies). All potential HF events and deaths were prospectively collected and adjudicated by blinded clinical endpoint committees.

### Statistical analysis

We first estimated the aggregate relative effects of comprehensive medical therapy based on individual treatment effects observed in each component trial. We used established methods of indirect comparisons, which are commonly applied in putative placebo assessments^30^. The accompanying 95% CI was estimated by the square root of the sum of the squared standard errors of the logarithmic HRs of the individual trial treatment effects. Cox proportional hazards models were used to estimate all time-to-first composite endpoints with trial-specific stratification terms as prespecified in each trial protocol: DELIVER (diabetes status)^3^, FINEARTS-HF (geographic region and LVEF <60% or ≥60%)^4^, and PARAGON-HF (geographic region)^5^. No covariate adjustment was made.

We considered comprehensive medical therapy as the combined use of SGLT2i and nsMRA for the overall population. As ARNI is indicated in many countries worldwide specifically for the treatment of patients with HF and LVEF below normal, we considered comprehensive medical therapy as the combined use of SGLT2i, nsMRA and ARNI for patients with an LVEF <60%. As PARAGON-HF was an active-controlled trial, we used the same methods of indirect comparisons to estimate the treatment effects of ARNI if it was instead compared against a putative placebo^30^. To do so, participant-level data were accessed from the CHARM-Preserved (Candesartan Cilexietil in Heart Failure Assessment of Reduction in Mortality and Morbidity) trial, which tested the angiotensin receptor blocker, candesartan (target dose 32 mg once daily) against placebo among 3,023 patients with symptomatic HF and LVEF >40% (ref. ^28^). As urgent HF visits were not collected or adjudicated in CHARM-Preserved, the endpoint of cardiovascular death or HF hospitalization was considered instead. We additionally conducted alternative segmenting aligned with contemporary guideline designations of LVEF^9,10^ and separately evaluated HFmrEF (LVEF between 40% and 50%) and HFpEF (LVEF of 50% or greater). The comparator populations were individuals treated according to the standard of care based on local guidelines, but did not mandate any specific pharmacotherapy.

We then projected the long-term absolute event-free survival gains by applying the imputed treatment effects of comprehensive medical therapy to the control group of the DELIVER trial. To consider individuals untreated with these therapies, we excluded individuals already treated with an ARNI or an MRA at baseline (*n* = 1,378). We leveraged validated actuarial (age-based) methods^18,19^ that reshape the follow-up horizon from considering time since randomization to evaluating age instead. At every age between 55 years and 85 years, we calculated nonparametric Kaplan–Meier estimates of residual survival free from the primary endpoint. Projected event-free survival was then estimated as the area under the survival curve (up to a maximum of 100 years). We separately estimated long-term event-free survival as observed in individuals in the DELIVER control arm (comparator group) and simulated if treated with comprehensive medical therapy. As there were no observed age-by-treatment interactions in any of the component trials^31–35^, the difference in areas under the survival curves represented the gains in event-free survival with comprehensive medical therapy at any given age of starting therapy. We additionally applied the lower and upper bounds of the 95% CI around the relative treatment effects to the DELIVER control group to provide a range of uncertainty of our estimates. For display purposes, event-free survival gains across the age range were smoothed after application of a locally weighted scatterplot smoothing procedure.

### Sensitivity analyses

We conducted a series of sensitivity and supplemental analyses to test the robustness of our cross-trial analysis. First, instead of considering DELIVER data alone, data from a meta-analysis of the two large SGLT2i outcomes trials in HFmrEF/HFpEF were used instead to summarize treatment effects of SGLT2i^32^. Similarly, instead of using FINEARTS-HF alone, data from a meta-analysis of FINEARTS-HF and a previous large HFmrEF/HFpEF outcomes trial of the steroidal MRA spironolactone were used instead to summarize treatment effects of MRA^33^. Second, we estimated the treatment effects of comprehensive medical therapy without making the assumption that two therapies when used together would provide fully additive effects. To do so, we assumed that the treatment effects of nsMRA may be 50%, 75% and 90% of its full efficacy when added to an SGLT2i. In each scenario, we multiplied the beta coefficient of the HR for nsMRA by the percentage of subadditive assumed effect; the resulting estimates were then inputted into the indirect comparison calculations to derive the treatment effects of comphrensive therapy. Third, we evaluated the long-term event-free survival gains of comprehensive medical therapy assuming declining or waning efficacy over time. Specifically, we assumed a yearly decline of 2%, 5% and 10% (compared with the previous year) in the efficacy of comprehensive medical therapy. Fourth, to account for potential competing risks of mortality, we evaluated comprehensive treatment effects on the composite endpoint of all-cause death or worsening HF event. Finally, to address concerns that urgent HF visits may not be as clinically meaningful as the other components of the composite endpoint, we evaluated a modified composite of cardiovascular death or hospitalization for HF, excluding urgent HF visits. Statistical analyses were performed using STATA, and *P* values less than 0.05 were considered statistically significant.

### Reporting summary

Further information on research design is available in the Nature Portfolio Reporting Summary linked to this article.

## Online content

Any methods, additional references, Nature Portfolio reporting summaries, source data, extended data, supplementary information, acknowledgements, peer review information; details of author contributions and competing interests; and statements of data and code availability are available at 10.1038/s41591-025-04037-3.

## Supplementary information

Reporting Summary

## Figures and Tables

**Extended Data Table 1:**
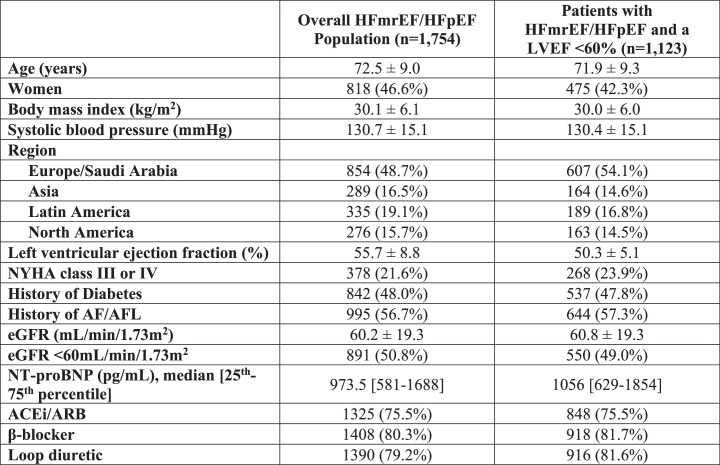
Baseline characteristics of base population from the DELIVER trial

**Extended Data Table 2:**
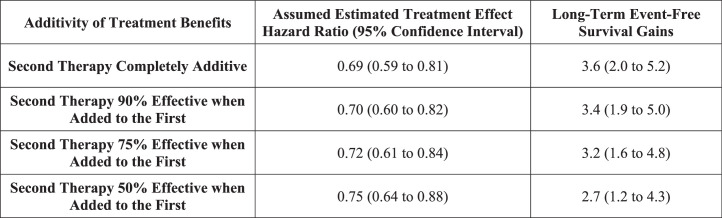
Sensitivity analysis assuming subadditive effects of comprehensive medical therapy

**Extended Data Table 3:**
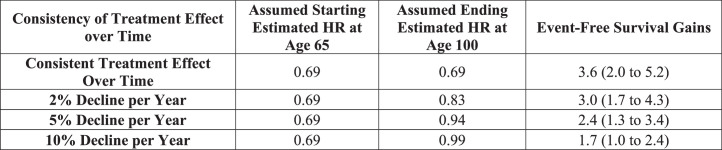
Sensitivity analysis assuming declining efficacy of comprehensive medical therapy over time

**Extended Data Table 4:**
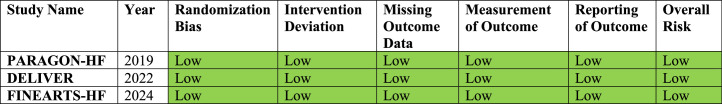
Risk of bias assessment using the revised tool to assess risk of bias in randomized trials (RoB 2.0)

## Data Availability

All trial funders are committed to sharing access to patient-level data and supporting clinical documents from eligible studies. Trial data availability is subject to the separate criteria and processes of AstraZeneca (https://astrazenecagrouptrials.pharmacm.com/ST/Submission/Disclosure), Bayer (https://vivli.org/ourmember/bayer/) and Novartis (https://www.novartis.com/sites/novartis_com/files/clinical-trial-data-transparency.pdf).
