# Supplementary material for: Lifetime benefits of comprehensive medical therapy in heart failure with mildly reduced or preserved ejection fraction
Source: Nat Med. 2025 Oct 6;32(1):325–31. doi: 10.1038/s41591-025-04037-3 (PMC12823412; doi:10.1038/s41591-025-04037-3)
Supplement: Supplementary file 1 — Reporting Summary [file 41591_2025_4037_MOESM1_ESM.pdf]

nature portfolio

Corresponding author(s): Scott D. Solomon, MD

Last updated by author(s): Sep 23, 2025

Reporting Summary

Nature Portfolio wishes to improve the reproducibility of the work that we publish. This form provides structure for consistency and transparency in reporting. For further information on Nature Portfolio policies, see our Editorial Policies and the Editorial Policy Checklist.

Statistics

For all statistical analyses, confirm that the following items are present in the figure legend, table legend, main text, or Methods section.

n/a	Confirmed	
☐	☒	The exact sample size (*n*) for each experimental group/condition, given as a discrete number and unit of measurement
☒	☐	A statement on whether measurements were taken from distinct samples or whether the same sample was measured repeatedly
☐	☒	The statistical test(s) used AND whether they are one- or two-sided<br>*Only common tests should be described solely by name; describe more complex techniques in the Methods section.*
☐	☒	A description of all covariates tested
☐	☒	A description of any assumptions or corrections, such as tests of normality and adjustment for multiple comparisons
☐	☒	A full description of the statistical parameters including central tendency (e.g. means) or other basic estimates (e.g. regression coefficient) AND variation (e.g. standard deviation) or associated estimates of uncertainty (e.g. confidence intervals)
☐	☒	For null hypothesis testing, the test statistic (e.g. *F*, *t*, *r*) with confidence intervals, effect sizes, degrees of freedom and *P* value noted<br>*Give P values as exact values whenever suitable.*
☒	☐	For Bayesian analysis, information on the choice of priors and Markov chain Monte Carlo settings
☒	☐	For hierarchical and complex designs, identification of the appropriate level for tests and full reporting of outcomes
☒	☐	Estimates of effect sizes (e.g. Cohen's *d*, Pearson's *r*), indicating how they were calculated

Our web collection on statistics for biologists contains articles on many of the points above.

Software and code

Policy information about availability of computer code

Data collection	No specific additional software was used for data collection for this cross-trial analysis.
Data analysis	Statistical analyses were conducted using STATA version 18.

For manuscripts utilizing custom algorithms or software that are central to the research but not yet described in published literature, software must be made available to editors and reviewers. We strongly encourage code deposition in a community repository (e.g. GitHub). See the Nature Portfolio guidelines for submitting code & software for further information.

Data

Policy information about availability of data

All manuscripts must include a data availability statement. This statement should provide the following information, where applicable:

- Accession codes, unique identifiers, or web links for publicly available datasets
- A description of any restrictions on data availability
- For clinical datasets or third party data, please ensure that the statement adheres to our policy

Individual participant data from the 3 completed trials were accessed. Categorical variables were harmonised and all variables computed to the same scale or units of measurement. Variable names were standardized across individual trial datasets. The baseline characteristics of trial participants were extracted and relevant subgroup variables extracted. Time to event (harmonized to days since randomization) and censoring variables for each of the outcomes listed were also extracted.

For each of the 3 clinical trials (DELIVER, FINEARTS-HF, and PARAGON-HF), the trial funders are committed to sharing access to patient-level data and supporting clinical documents from eligible studies. The trial data availability is according to the separate criteria and processes for AstraZeneca (https://astrazenecagrouptrials.pharmacm.com/ST/Submission/Disclosure), Bayer (https://vivli.org/ourmember/bayer/), and Novartis (https://www.novartis.com/sites/novartis_com/files/clinical-trial-data-transparency.pdf).

Research involving human participants, their data, or biological material

Policy information about studies with human participants or human data. See also policy information about sex, gender (identity/presentation), and sexual orientation and race, ethnicity and racism.

Reporting on sex and gender	The n (%) of women was described by trial population (DELIVER, FINEARTS-HF, and PARAGON-HF).
Reporting on race, ethnicity, or other socially relevant groupings	This report does not contain any specific race, ethnicity, or socially relevant group data.
Population characteristics	Patients with heart failure with mildly reduced or preserved ejection fraction
Recruitment	All participants randomized in each of the 3 trials were considered for this cross-trial analysis with only patients with critical Good Clinical Practice violations excluded.
Ethics oversight	The trial protocols were approved by ethics committees or institutional review boards at all participating sites and all patients provided explicit written informed consent.

Note that full information on the approval of the study protocol must also be provided in the manuscript.

Field-specific reporting

Please select the one below that is the best fit for your research. If you are not sure, read the appropriate sections before making your selection.

☒ Life sciences ☐ Behavioural & social sciences ☐ Ecological, evolutionary & environmental sciences

For a reference copy of the document with all sections, see nature.com/documents/nr-reporting-summary-flat.pdf

Life sciences study design

All studies must disclose on these points even when the disclosure is negative.

Sample size	For the main analysis, we derived treatment estimates from 6,263 participants in DELIVER and 6,001 participants in FINEARTS-HF. In the subgroup of individuals with LVEF below normal (<60%), we estimated treatment effects from 4,372 participants in DELIVER, 4,846 in FINEARTS-HF, and 2,070 in PARAGON-HF. As this was a cross-trial analysis of completed randomized clinical trials, we considered all randomized participants under intention-to-treat principles. These samples represent the largest trials evaluating each individual drug therapy in this target population and thus are felt to sufficient to provide stable estimates of treatment effects on clinical outcomes.
Data exclusions	Only patients with critical Good Clinical Practice violations were excluded.
Replication	As randomized assessments of comprehensive therapy have not been conducted to date in this target population, replication of our cross-trial analysis was not feasible.
Randomization	In the DELIVER trial, 6,263 adults ≥40 years with symptomatic HF and an LVEF>40% were randomly assigned 1:1 to dapagliflozin 10mg once daily or matching placebo. In the FINEARTS-HF trial, 6,001 adults ≥40 years with symptomatic HF and an LVEF ≥40% were randomly assigned 1:1 to finerenone or matching placebo titrated to target doses of 20mg or 40mg (depending on baseline estimated glomerular filtration rate). In PARAGON-HF, 4,796 adults ≥18 years with symptomatic HF and an LVEF ≥45% were randomly assigned to the ARNI sacubitril/valsartan (target dose, 97 mg of sacubitril with 103 mg of valsartan twice daily) versus the angiotensin receptor blocker valsartan (target dose, 160 mg twice daily). Only participants who tolerated half target doses of both study medications during a single-blind run-in phase were randomized.
Blinding	All 3 trials were double-blind randomized clinical trials. Specifically, all investigators and participants remained strictly blinded to treatment arm allocation during the randomized period.

Reporting for specific materials, systems and methods

We require information from authors about some types of materials, experimental systems and methods used in many studies. Here, indicate whether each material, system or method listed is relevant to your study. If you are not sure if a list item applies to your research, read the appropriate section before selecting a response.

Materials & experimental systems

n/a	Involved in the study
☒	☐ Antibodies
☒	☐ Eukaryotic cell lines
☒	☐ Palaeontology and archaeology
☒	☐ Animals and other organisms
☐	☒ Clinical data
☒	☐ Dual use research of concern
☒	☐ Plants

Methods

n/a	Involved in the study
☒	☐ ChIP-seq
☒	☐ Flow cytometry
☒	☐ MRI-based neuroimaging

Clinical data

Policy information about clinical studies

All manuscripts should comply with the ICMJE guidelines for publication of clinical research and a completed CONSORT checklist must be included with all submissions.

Clinical trial registration: DELIVER (Dapagliflozin Evaluation to Improve the LIVEs of Patients with Preserved Ejection Fraction Heart Failure; ClinicalTrials.gov Identifier: NCT03619213); FINEARTS-HF (FINerenone trial to investigate Efficacy and sAfety superioR to placebo in paTientS with Heart Failure; ClinicalTrials.gov Identifier: NCT04435626), PARAGON-HF (Prospective Comparison of ARNI [angiotensin receptor–neprilysin inhibitor] with ARB Global Outcomes in HF with Preserved Ejection Fraction; ClinicalTrials.gov Identifier: NCT01920711)

Study protocol: The study protocols and the statistical analysis plans for each of the included trials are published and publicly available for review

Data collection: Participants in DELIVER were enrolled from 2018 to 2021 across 20 countries. Participants in FINEARTS-HF were enrolled from 2020 through 2023 across 37 countries. Participants in PARAGON-HF were enrolled from 2014 to 2016 across 43 countries. All 3 trials were global clinical trials with enrollment from academic/hospital-based or community health care facilities.

Outcomes: The primary endpoint was a composite of cardiovascular death or worsening HF event (which included both hospitalizations for HF and urgent ambulatory encounters for HF requiring intravenous HF therapies). All potential HF events and deaths were prospectively collected and adjudicated by blinded clinical endpoints committees.

Plants

Seed stocks: *Report on the source of all seed stocks or other plant material used. If applicable, state the seed stock centre and catalogue number. If plant specimens were collected from the field, describe the collection location, date and sampling procedures.*

Novel plant genotypes: *Describe the methods by which all novel plant genotypes were produced. This includes those generated by transgenic approaches, gene editing, chemical/radiation-based mutagenesis and hybridization. For transgenic lines, describe the transformation method, the number of independent lines analyzed and the generation upon which experiments were performed. For gene-edited lines, describe the editor used, the endogenous sequence targeted for editing, the targeting guide RNA sequence (if applicable) and how the editor was applied.*

Authentication: *Describe any authentication procedures for each seed stock used or novel genotype generated. Describe any experiments used to assess the effect of a mutation and, where applicable, how potential secondary effects (e.g. second site T-DNA insertions, mosiacism, off-target gene editing) were examined.*
